# Editorial: Ethnopharmacological Responses to the Coronavirus Disease 2019 Pandemic

**DOI:** 10.3389/fphar.2021.798674

**Published:** 2021-12-03

**Authors:** Jia-bo Wang, Adolfo Andrade-Cetto, Javier Echeverria, Jon Wardle, Hung-Rong Yen, Michael Heinrich

**Affiliations:** ^1^ School of Traditional Chinese Medicine, Capital Medical University, Beijing, China; ^2^ Laboratorio de Etnofarmacología, Facultad de Ciencias, Universidad Nacional Autónoma de México, Mexico City, Mexico; ^3^ Departamento de Ciencias del Ambiente, Facultad de Química y Biología, Universidad de Santiago de Chile, Santiago, Chile; ^4^ National Centre for Naturopathic Medicine, Southern Cross University, Lismore, NSW, Australia; ^5^ Chinese Medicine Research Center and College of Chinese Medicine, China Medical University, Taichung, Taiwan; ^6^ Research Group “Pharmacognosy and Phytotherapy”, UCL School of Pharmacy, University of London, London, United Kingdom

**Keywords:** COVID-19, ethnopharmacology, traditional medicine, adjuvant therapies, clinical study, systematic review, mechanism study

## 1 Introduction

The pandemic of coronavirus disease 2019 (COVID-19), caused by severe acute respiratory syndrome coronavirus 2 (SARS-CoV-2) infection, has led to severe impact on health globally. There is no effective therapy for SARS-CoV-2 infection except the preventive effects of a vaccine. Several medicines (PF-07321332, EXO-CD24, BRII-196, and BRII-198 etc.) against SARS-CoV-2 infection are under development; but the clinical efficacy of these medicines has not been evaluated rigorously. The outputs and prices make them less accessible, especially in low-and middle-income countries (LMIC). The lack of effective medicines has led to the very high global demand for various forms of traditional medicines (TM) as a potential treatment option for COVID-19. Simultaneously, the use of herbal medicines and supplements (especially those with anti-infective and immunomodulatory effects as well as those used as a supportive therapy) has increased dramatically as a part of adjuvant therapy in economically developed countries, which is often not reported to health care professionals (Bhamra et al. 2021; Smith et al.). In many LMIC regions including in China, India, South Korea, Thailand, the Americas and Africa, TMs are accessible and widely used and numerous TM therapies have started to be investigated as potential treatments. Some of the claims made, however, are very high and seem implausible. We reiterate that these TM treatments should be seen as a part of a broader package of adjuvant and symptomatic treatments and that scientists have a specific responsibility to ascertain a balanced and critical assessment of the evidence as well as the gaps (Heinrich 2010).

For example, traditional African medicine (TAM), traditional Chinese medicine (TCM), traditional Indian medicine (TIM), and traditional Persian medicine (TPM) have shown unique potentials to be used generally as a therapy against COVID-19. This is linked to reported immunomodulatory, anti-inflammatory, antiviral, antioxidant, antihistamine, and bronchodilator effects (Bahramsoltani and Rahimi; Ahmad et al.; Attah et al.; Mosleh et al.; Saggam et al.). This widespread use and the existing preliminary evidence highlight the need of clinical evidence from rigorous randomized controlled trials (RCTs) and meta-analysis, as well as *in vitro* and *in vivo* studies to assess the efficacy and the mechanisms of TMs against COVID-19.

This research topic is a collection of 37 articles focusing on the adjuvant effects, pharmacological characteristics, efficacy and safety of TMs, and the potential mechanisms against COVID-19 of TM formulations, medicinal plants, and active components.

## 2 Usage of TMs in the Treatment of COVID-19 and Related Symptoms

**FIGURE 1:**
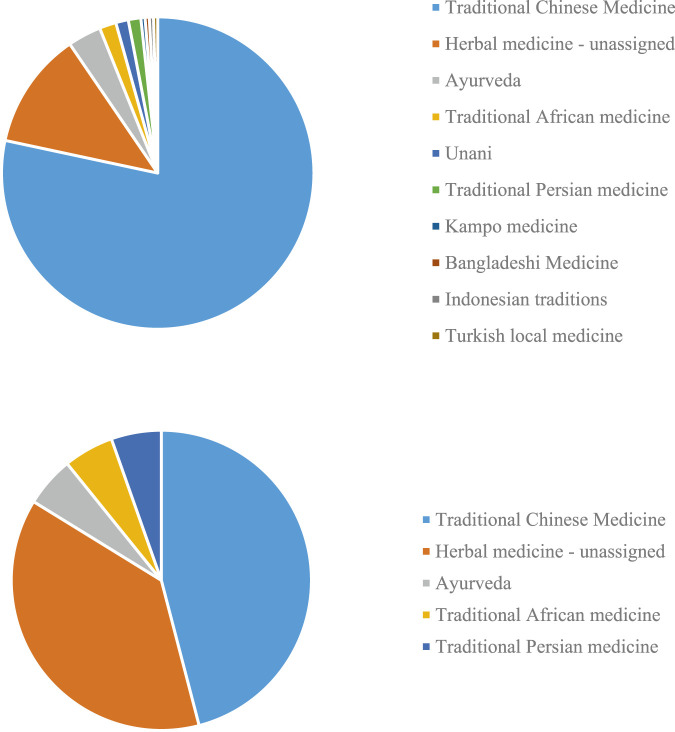
COVID-19 papers focusing on different local and traditional medical systems (TMs) **(A)** in PubMed. and **(B)** represented in this research topic.

To get a view of the usage of TMs in the treatment of COVID-19, we searched PubMed to illustrate the proportion of different kinds of TMs with the time range from January 1, 2019 to August 12, 2021. In total, 231 studies of TMs on COVID-19 were retrieved. Among these studies, most abundant were studies on TCM (nearly 80%; 181/231); followed by studies which could not be classified into a specific geographical region or traditional medicine system (28 studies) (cf. also Brendler et al., 2021). The rest of the studies covered traditional medicine systems Ayurveda (Indian medicine), traditional, African, Unani, Kampo, and medicines from Bangladesh, Iran, Indonesia and Turkey (Figure 1A) (Ai et al., 2020; Ayatollahi et al., 2021; Benarba and Pandiella, 2020; Chen et al., 2021; Fakhri et al., 2021; Ge and He, 2020; Jha et al., 2021; Kim, 2021; Kalhori et al., 2021; Lem et al., 2021; Li et al., 2020; Lim et al., 2021; Liu et al., 2020; Luo et al., 2020; Majnooni et al., 2020; Ma et al., 2021; Mandal et al., 2021; Peter et al., 2021; Qiu et al., 2020; Remali and Aizat, 2021; Ren et al., 2021; Silveira et al., 2020; Spicer, 2021; Verma et al., 2020; Wu et al., 2020; Wu et al., 2021; Yang et al., 2020; Zhang et al., 2020; Zhou et al., 2020).

This research topic includes research on TCM, herbal medicine generally, Ayurvedic medicine, African, and Iranian medical traditions (Figure 1B), with TCM studies accounting for nearly 50% (17/37).

## 3 Evidence-Based Medicine of TM Studies in the Treatment of COVID-19

**FIGURE 2:**
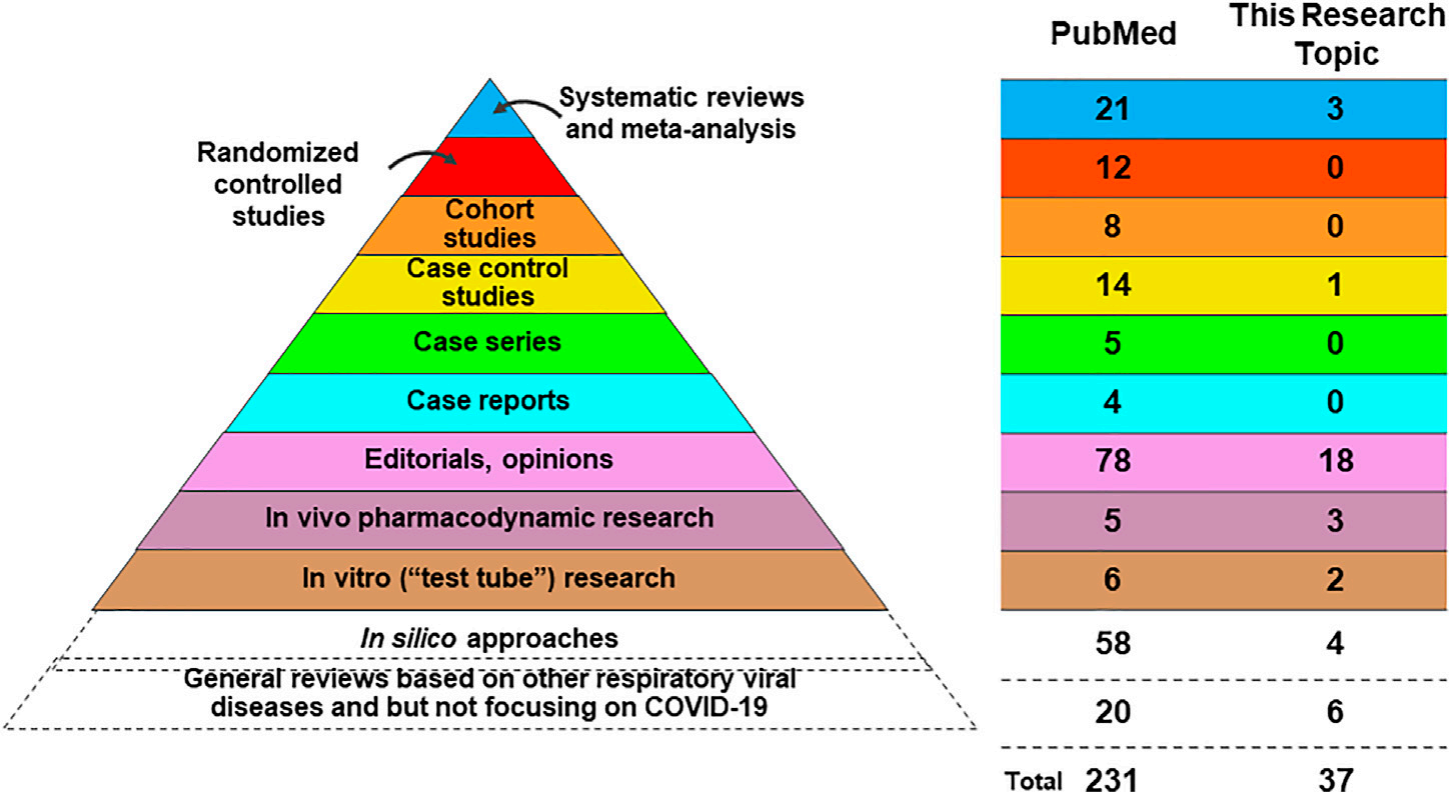
Publication on TM associated with COVID-19.

Rigorous assessment on the level of evidence is of critical importance to guide future research and clinical management on COVID-19 by TMs. Looking at the top level of evidence regarding the study of TMs on COVID-19 (Figure 2), in PubMed there are 21 systematic reviews (SRs) and meta-analyses and 3 SRs in this research topic. In PubMed 12 randomized controlled trials (RCTs) are recorded, and 11 of these RCTs evaluated the efficacy and safety of TCM in the treatment of COVID-19 and the other RCT (published on Frontiers in Pharmacology but not this research topic), tested oral use of a combined curcumin and piperine tablet, which are commonly used in India. We noted that we have no RCT papers but three high-quality SRs in this research topic. Two of these three SRs performed meta-analysis and synthesized the current evidence to evaluate the clinical and safety of TCM intervention in the treatment of COVID-19. Beyond these RCT studies and meta-analysis, however, we can see that the majority of TMs-related COVID-19 studies indicate a relatively low level of evidence. There remains a dearth of original research on this topic, and those opinion papers or descriptive reviews have much overlap of ideas and cited references resulting in the risk of a limited scientific impact. With the limitation of good *in vivo* and *in vitro* SARS-Cov-2 models, we found a very limited body of scientific evidence. However, we found many *in silico* papers using molecular docking or network pharmacology that offer hypothetic leads based on TMs. Such non-validated *in silico* studies on TMs are, without associated *in vivo*, *in vitro* or clinical evidence, of limited relevance for informing clinical practice in COVID-19 and can easily be misleading. Some review papers have been written based on experiences with other respiratory viral diseases but not based on COVID-19, specifically. These review papers were usually published in the early period of the pandemic, with a very limited knowledge regarding to COVID-19 at the time. These reviews may inspire new ideas for future studies on COVID-19, or of future management of a pandemic, considering the potential similarities of corona viruses and common courses of respiratory diseases and inflammatory responses. For instance, TCM treatments were broadly used and played an important role in other respiratory diseases, such as the severe acute respiratory syndrome (SARS) in 2003, the Influenza A (H1N1) in 2009, the Middle East Respiratory Syndrome (MERS) in 2012, and H7N9 avian influenza in 2013 (Xi et al.; Zhuang et al.), which may have some inspirations to understand the treatment of COVID-19. Collectively, although there is a growing body of publications on TMs against COVID-19, we need to be aware that we still lack high-quality clinical evidence, which highlights the importance of future clinical studies.

## 4 Emphasis on the Clinical Evidences of TMs in the Treatment of COVID-19

Two SRs in this research topic evaluated the clinical efficacy of TCM in the treatment of COVID-19 and concluded that the interventions were safe in COVID-19 patients (Liang et al.; Wang et al.). One of these illustrated that TCM in combination with conventional therapy was better than conventional therapy alone. The purported beneficial effects included increasing the recovery rate of main symptoms of cough and fatigue, shortening the duration of main symptoms of fever, cough and fatigue, but were not suggestive of an increase the recovery rate of main symptoms of fever (Liang et al.). Another SR and meta-analysis focusing on low-risk-of-bias RCTs showed moderate confidence that compare to routine treatment alone, TCM plus routine treatment could reduce the incidence of unfavourable events of clinical deterioration, acute respiratory distress syndrome (ARDS), mechanical ventilation, and death). However, the treatment was not found to reduce the level of positive tests using the SARS-CoV-2 nucleic acid test, and on chest X-ray images could shows improvements based (Wang et al.). Considering ARDS is the most common complications of COVID-19 (about 7.4–41.8% of COVID-19 patients developed ARDS), as well as one of the most dangerous (the mortality rate of COVID-19 patients with ARDS was 30.4–52.4%), the potentially favourable effect of specific TCM formula may indicate significant merit in further mechanistic studies (Wang et al.).

## 5 Perspectives

TMs have been used extensively in preventing and treating COVID-19 worldwide and in this short period a significant number papers have been published including a considerable share in this RT. These publications attracted considerable attention and have impacted on the fast-evolving discussion about the use of TMs. A wider debate is about the future role of TMs. Some countries including Thailand and the PR China have embraced TM as a potential strategy for ongoing treatment and/or prevention of COVID-19. In some cases, very strong claims about what can be achieved have been made, and these too may warrant further research attention. In many countries, however, the use of TMs as a strategy for COVID-19 is not accepted, resulting in such treatments becoming limited to over-the-counter self-treatment, often in unregulated or informal settings. An example is the dramatic rise of elderberry-based products (*Sambucus nigra* L.) sold in the United States in 2020 (Smith et al., 2021). Here the systematic assessment of opportunities to use such treatments as adjuvants and their appropriate role in the self-management of respiratory conditions more generally needs to be developed further. However, we should keep in mind to the lack of high-quality evidence-based TM-based treatments of COVID-19. More importantly, although many kinds of popular medicines have been used in the management of COVID-19, so far only a few TCM preparations appear to have been tested systematically in RCTs, and even then these studies remain small and of uncertain quality. High quality clinical studies are urgently needed to appropriately guide evidence-informed approaches to incorporation of TMs in public health systems responses to COVID-19. Research using *in silico* or *in vitro* methods may have some values but the results need to be investigated further *in vivo* and clinically in order to allow an assessment of potential therapeutic benefits. Another limitation on the research on TMs against COVID-19 is the limited access to appropriate animal models, for example, transgenic hACE2 mice model (Bao et al., 2020) and cytokine storm syndrome mouse model induced by SARS-CoV-2 Spike protein (Gu et al., 2021). On the other hand, long COVID, the post-acute COVID-19 syndrome, has emerged as a major concern, which deserves further studies by using TMs (Adeloye et al., 2021). Overall, this research topic has been one of the first responses of the scientific communities globally to assess the potential of TM and here specifically TMs in addressing this major global health challenge. It has demonstrated the enormous importance and potential of TMs globally in response to the pandemic and the fast evolving scientific evidence base for some of these treatments. However, it also serves as a calling for a much more systematic study of TMs globally including, obviously, the need for major funding of this research, in order to be appropriately informed on this topic.

## References

[B1] Adeloye D. Elneima O. Daines L. Poinasamy K. Quint J. K. Walker S. (2021). The Long-Term Sequelae of COVID-19: an International Consensus on Research Priorities for Patients with Pre-existing and New-Onset Airways Disease. Lancet Respir. Med., 34416191. 10.1016/S2213-2600(21)00286-1 PMC8372501 34416191

[B2] Ai Z. Zhou S. Li W. Wang M. Wang L. Hu G. (2020). "Fei Yan No. 1" as a Combined Treatment for COVID-19: An Efficacy and Potential Mechanistic Study. Front. Pharmacol. 11, 581277. 10.3389/fphar.2020.581277 33132913 PMC7580177

[B3] Ayatollahi S. A. Sharifi-Rad J. Tsouh Fokou P. V. Mahady G. B. Ansar Rasul Suleria H. Krishna Kapuganti S. (2021). Naturally Occurring Bioactives as Antivirals: Emphasis on Coronavirus Infection. Front. Pharmacol. 12, 575877. 10.3389/fphar.2021.575877 34267652 PMC8277242

[B4] Bao L. Deng W. Huang B. Gao H. Liu J. Ren L. (2020). The Pathogenicity of SARS-CoV-2 in hACE2 Transgenic Mice. Nature 583, 830–833. 10.1038/s41586-020-2312-y 32380511

[B5] Benarba B. Pandiella A. (2020). Medicinal Plants as Sources of Active Molecules against COVID-19. Front. Pharmacol. 11, 1189. 10.3389/fphar.2020.01189 32848790 PMC7427466

[B6] Bhamra S. K. Parmar J. Heinrich M. (2021). The COVID-19 Pandemic and its Impact on the Professional Practice and Personal Wellbeing of Community Pharmacy Teams in the UK. Int. J. Pharm. Pract., riab062. 10.1093/ijpp/riab062 PMC8500076 34605895

[B7] Brendler T. Al-Harrasi A. Bauer R. Gafner S. Hardy M. L. Heinrich M. (2021). Botanical Drugs and Supplements Affecting the Immune Response in the Time of COVID-19: Implications for Research and Clinical Practice. Phytother Res. 35 (6), 3013–3031. Epub 2020 Dec 29. PMID: 33373071. 10.1002/ptr.7008 33373071

[B8] Chen R. H. Yang L. J. Hamdoun S. Chung S. K. Lam C. W. Zhang K. X. (2021). 1,2,3,4,6-Pentagalloyl Glucose, a RBD-ACE2 Binding Inhibitor to Prevent SARS-CoV-2 Infection. Front. Pharmacol. 12, 634176. 10.3389/fphar.2021.634176 33897423 PMC8058605

[B9] Fakhri S. Piri S. Majnooni M. B. Farzaei M. H. Echeverría J. (2020). Targeting Neurological Manifestations of Coronaviruses by Candidate Phytochemicals: A Mechanistic Approach. Front. Pharmacol. 11, 621099. 10.3389/fphar.2020.621099 33708124 PMC7941749

[B10] Ge C. He Y. (2020). In Silico Prediction of Molecular Targets of Astragaloside IV for Alleviation of COVID-19 Hyperinflammation by Systems Network Pharmacology and Bioinformatic Gene Expression Analysis. Front. Pharmacol. 11, 556984. 10.3389/fphar.2020.556984 33041797 PMC7525161

[B11] Gu T. Zhao S. Jin G. Song M. Zhi Y. Zhao R. (2021). Cytokine Signature Induced by SARS-CoV-2 Spike Protein in a Mouse Model. Front. Immunol. 11, 621441. 10.3389/fimmu.2020.621441 33584719 PMC7876321

[B12] Heinrich M. (2010). Ethnopharmacology in the 21st century - Grand Challenges. Front. Pharmacol. 1, 8. 10.3389/fphar.2010.00008 21713103 PMC3112271

[B13] Jha N. K. Sharma C. Hashiesh H. M. Arunachalam S. Meeran M. N. Javed H. (2021). β-Caryophyllene, A Natural Dietary CB2 Receptor Selective Cannabinoid Can Be a Candidate to Target the Trinity of Infection, Immunity, and Inflammation in COVID-19. Front. Pharmacol. 12, 590201. 10.3389/fphar.2021.590201 34054510 PMC8163236

[B14] Kalhori M. R. Saadatpour F. Arefian E. Soleimani M. Farzaei M. H. Aneva I. Y. (2021). The Potential Therapeutic Effect of RNA Interference and Natural Products on COVID-19: A Review of the Coronaviruses Infection. Front. Pharmacol. 12, 616993. 10.3389/fphar.2021.616993 33716745 PMC7953353

[B15] Kim C. H. (2021). Anti-SARS-CoV-2 Natural Products as Potentially Therapeutic Agents. Front. Pharmacol. 12, 590509. 10.3389/fphar.2021.590509 34122058 PMC8194829

[B16] Lem F. F. Opook F. Lee D. J. H. Chee F. T. Lawson F. P. Chin S. N. (2020). Molecular Mechanism of Action of Repurposed Drugs and Traditional Chinese Medicine Used for the Treatment of Patients Infected with COVID-19: A Systematic Scoping Review. Front. Pharmacol. 11, 585331. 10.3389/fphar.2020.585331 33746739 PMC7970521

[B17] Li Q. Bai C. Yang R. Xing W. Pang X. Wu S. (2020). Deciphering the Pharmacological Mechanisms of Ma Xing Shi Gan Decoction against COVID-19 through Integrating Network Pharmacology and Experimental Exploration. Front. Pharmacol. 11, 581691. 10.3389/fphar.2020.581691 33324213 PMC7725906

[B18] Lim X. Y. Teh B. P. Tan T. Y. C. (2021). Medicinal Plants in COVID-19: Potential and Limitations. Front. Pharmacol. 12, 611408. 10.3389/fphar.2021.611408 33841143 PMC8025226

[B19] Liu T. Guo Y. Zhao J. He S. Bai Y. Wang N. (2020). Systems Pharmacology and Verification of ShenFuHuang Formula in Zebrafish Model Reveal Multi-Scale Treatment Strategy for Septic Syndrome in COVID-19. Front. Pharmacol. 11, 584057. 10.3389/fphar.2020.584057 33041827 PMC7523021

[B20] Luo C. H. Ma L. L. Liu H. M. Liao W. Xu R. C. Ci Z. M. (2020). Research Progress on Main Symptoms of Novel Coronavirus Pneumonia Improved by Traditional Chinese Medicine. Front. Pharmacol. 11, 556885. 10.3389/fphar.2020.556885 33013395 PMC7516165

[B21] Ma L. L. Liu H. M. Luo C. H. He Y. N. Wang F. Huang H. Z. (2021). Fever and Antipyretic Supported by Traditional Chinese Medicine: A Multi-Pathway Regulation. Front. Pharmacol. 12, 583279. 10.3389/fphar.2021.583279 33828481 PMC8020597

[B22] Majnooni M. B. Fakhri S. Shokoohinia Y. Kiyani N. Stage K. Mohammadi P. (2020). Phytochemicals: Potential Therapeutic Interventions against Coronavirus-Associated Lung Injury. Front. Pharmacol. 11, 588467. 10.3389/fphar.2020.588467 33658931 PMC7919380

[B23] Mandal A. Jha A. K. Hazra B. (2021). Plant Products as Inhibitors of Coronavirus 3CL Protease. Front. Pharmacol. 12, 583387. 10.3389/fphar.2021.583387 33767619 PMC7985176

[B24] Peter A. E. Sandeep B. V. Rao B. G. Kalpana V. L. (2020). Calming the Storm: Natural Immunosuppressants as Adjuvants to Target the Cytokine Storm in COVID-19. Front. Pharmacol. 11, 583777. 10.3389/fphar.2020.583777 33708109 PMC7941276

[B25] Qiu Q. Huang Y. Liu X. Huang F. Li X. Cui L. (2020). Potential Therapeutic Effect of Traditional Chinese Medicine on Coronavirus Disease 2019: A Review. Front. Pharmacol. 11, 570893. 10.3389/fphar.2020.570893 33343347 PMC7741169

[B26] Remali J. Aizat W. M. (2020). A Review on Plant Bioactive Compounds and Their Modes of Action against Coronavirus Infection. Front. Pharmacol. 11, 589044. 10.3389/fphar.2020.589044 33519449 PMC7845143

[B27] Ren W. Ma Y. Wang R. Liang P. Sun Q. Pu Q. (2021). Research Advance on Qingfei Paidu Decoction in Prescription Principle, Mechanism Analysis and Clinical Application. Front. Pharmacol. 11, 589714. 10.3389/fphar.2020.589714 33584265 PMC7873690

[B28] Silveira D. Prieto-Garcia J. M. Boylan F. Estrada O. Fonseca-Bazzo Y. M. Jamal C. M. (2020). COVID-19: Is There Evidence for the Use of Herbal Medicines as Adjuvant Symptomatic Therapy? Front. Pharmacol. 11, 581840. 10.3389/fphar.2020.581840 33071794 PMC7542597

[B29] Smith T. Majid F. Eckl V. Morton Reynolds C. (2021). Herbal Supplement Sales in US Increase by Record-Breaking 17.3% in 2020. Herbal Gram. (131), 52–65. Available: http://herbalgram.org/resources/herbalgram/issues/131/table-of-contents/hg131-mkrpt/ .

[B30] Spicer D. (2021). Pilot Trial of XFBD, a TCM, in Persons with COVID-19. Available at: https://clinicaltrials.gov/ct2/show/NCT04810689 August 17, 2021).Assessed at

[B31] Verma S. Twilley D. Esmear T. Oosthuizen C. B. Reid A. M. Nel M. (2020). Anti-SARS-CoV Natural Products with the Potential to Inhibit SARS-CoV-2 (COVID-19). Front. Pharmacol. 11, 561334. 10.3389/fphar.2020.561334 33101023 PMC7546787

[B32] Wu C. Y. Lin Y. S. Yang Y. H. Shu L. H. Cheng Y. C. Liu H. T. (2020). Potential Simultaneous Inhibitors of Angiotensin-Converting Enzyme 2 and Transmembrane Protease, Serine 2. Front. Pharmacol. 11, 584158. 10.3389/fphar.2020.584158 33390952 PMC7773841

[B33] Wu J. Sun B. Hou L. Guan F. Wang L. Cheng P. (2020). Prospective: Evolution of Chinese Medicine to Treat COVID-19 Patients in China. Front. Pharmacol. 11, 615287. 10.3389/fphar.2020.615287 33716728 PMC7947616

[B34] Yang C. W. Lee Y. Z. Hsu H. Y. Jan J. T. Lin Y. L. Chang S. Y. (2020). Inhibition of SARS-CoV-2 by Highly Potent Broad-Spectrum Anti-coronaviral Tylophorine-Based Derivatives. Front. Pharmacol. 11, 606097. 10.3389/fphar.2020.606097 33519469 PMC7845692

[B35] Zhang X. R. Li T. N. Ren Y. Y. Zeng Y. J. Lv H. Y. Wang J. (2020). The Important Role of Volatile Components from a Traditional Chinese Medicine Dayuan-Yin against the COVID-19 Pandemic. Front. Pharmacol. 11, 583651. 10.3389/fphar.2020.583651 33101037 PMC7546797

[B36] Zhou Z. Gao N. Wang Y. Chang P. Tong Y. Fu S. (2020). Clinical Studies on the Treatment of Novel Coronavirus Pneumonia with Traditional Chinese Medicine-A Literature Analysis. Front. Pharmacol. 11, 560448. 10.3389/fphar.2020.560448 33013397 PMC7511712

